# The Co-Evolution of Fairness Preferences and Costly Punishment

**DOI:** 10.1371/journal.pone.0054308

**Published:** 2013-03-20

**Authors:** Moritz Hetzer, Didier Sornette

**Affiliations:** 1 Chair of Entrepreneurial Risks, Department of Management, Technology and Economics, Swiss Federal Institute of Technology, Zurich, Switzerland; 2 Swiss Finance Institute, Geneva, Switzerland; University of Massachusetts, United States of America

## Abstract

We study the co-evolutionary emergence of fairness preferences in the form of other-regarding behavior and its effect on the origination of costly punishment behavior in public good games. Our approach closely combines empirical results from three experiments with an evolutionary simulation model. In this way, we try to fill a gap between the evolutionary theoretical literature on cooperation and punishment on the one hand and the empirical findings from experimental economics on the other hand. As a principal result, we show that the evolution among interacting agents inevitably favors a sense for fairness in the form of “disadvantageous inequity aversion”. The evolutionary dominance and stability of disadvantageous inequity aversion is demonstrated by enabling agents to co-evolve with different self- and other-regarding preferences in a competitive environment with limited resources. Disadvantageous inequity aversion leads to the emergence of costly (“altruistic”) punishment behavior and quantitatively explains the level of punishment observed in contemporary lab experiments performed on subjects with a western culture. Our findings corroborate, complement, and interlink the experimental and theoretical literature that has shown the importance of other-regarding behavior in various decision settings.

## Introduction

Why do we show other-regarding or even altruistic behaviors? Why and how did we develop a sense for fairness? Is such behavior compatible with Darwin's principle of fitness maximization and/or with the economic axiom of rational decision making? Which evolutionary mechanisms dominate the evolution of our pro-sociality? This article aims at shedding light on the puzzling behavior of pro-sociality. The paper presents an approach to explain the emergence of fairness preferences and costly punishment behavior, which is motivated by perspectives from biology, evolutionary psychology, sociology and economics.

There is evidence from a variety of studies that fairness preferences have emerged in hominids over hundreds and thousands of years, with roots in our genetic heritage as evidence from recent studies on primates and the genetic encoding of social behavior suggests [1]–[9]. The importance of our genetic heritage for the structural basis of our pro-sociality appears to be plausible: Our genes encode the essential protein and RNA structures that are required to build up our physical-, cognitive- and computational capabilities. These capabilities allow us e.g. to perceive others' behavior, to compare quantities and to interact either physically or by communication with our environment. Furthermore, they build the fundamental basis that allows us to express, transmit and externalize our cumulative knowledge, our culture. Vice versa, our cultural evolution promotes those genes which are beneficial to the cultural evolution itself. Culture and genes thus appear to be subjected to more complex, co-evolutionary processes occurring over a spectrum of different time scales. Cultural evolution is shaped by biological conditions, while, simultaneously, genes are altered in response to the evolutionary forces induced by the cultural context. As a consequence, the perception of fairness and the reaction to unfair behavior as well as the individual's response to its social environment in general seem to be encoded both in cultural norms and in genes [10]–[16].

As an ultimate result, the coordination and convergence of individual attitudes to common group behavior and the emergence of social norms as well as their enforcement by informal social sanctions are often observed in groups of animals and human societies [17]–[21]. From small cliques to the social order in groups and tribes, all the way to the legal frameworks of countries, punishment is a widespread mechanism underlying the formation of social norms [22]–[24]. Various forms of punishment, ranging from symmetric peer punishment to asymmetric third party punishment, e.g. in criminal prosecutions, reflect enforcement mechanisms and are expressions of internalized norms and rules. In particular, costly punishment, i.e. the punishment of norm violators at one's own cost without personal benefit, is frequent in social dilemma experiments and is often used to explain the high level of cooperation between humans [24]–[29]. From an evolutionary perspective, natural selection should discriminate against altruistic individuals who incur costs to themselves in order to provide benefits to non-relatives and to strangers in one-shot interactions. Within Darwin's theory as well as in economic and game theoretical models, which rely on rational selfishness and the dominance of self-regarding preferences, such behaviors are puzzling, if not disrupting.

Models of kin selection (inclusive fitness), reciprocity with or without spatial and social structures (network reciprocity), group-level and multi-level selection have been developed to explain the presence of pro-social behavior [30]–[37]. Laboratory experiments and field studies suggest that egalitarian motives and other-regarding preferences, which relate a person's decision to her social environment, have a significant influence in social dilemmas, coordination and bargaining games [38]–[41]. As a result, psychological models of inequity aversion have been formulated that included descriptions of other-regarding preferences. These models are based on motivation functions that include relative income preferences, envy, inequality aversion and altruism [42]–[45].

The quantitative comparison with empirical data often remains unsatisfactory as most models aim at explaining stylized facts rather than providing quantitative explanations of the generating mechanisms. Therefore, it also remains vague on what the exact nature of our preferences and behavior should be. While based on plausible assumptions, an evolutionary validation of these assumptions is not manifested.

This paper addresses the question whether and under what conditions other-regarding preferences can emerge, evolve and ultimately dominate pure self-regarding and selfish behavior and, consequently, whether the presence of other-regarding preferences can cause and preserve altruistic feedback mechanisms such as costly punishment. The lack of a sound connection between the literature concerned with the evolution of cooperation and the experimental economics literature has created intense discussions and various interpretations on how our pro-social behavior is shaped and what the field studies and lab experiments show and do not show [29], [46]–[54].

The present paper aims at filling the gap between the theoretical literature on the evolution of cooperation and punishment, and the empirical findings from experimental economics. Thereby it borrows ideas from evolutionary biology, behavioral sciences and -economics as well as complex system science.

Experiments on public goods and social dilemma games provide convenient tools to study social preferences in well-defined scenarios under controlled conditions. In these experiments, one can study in details what controls the predisposition of humans to bear the costs associated with punishment of free riders, and how it may improve the welfare of the group. The observed behavior in the experiments can be interpreted as sampling the statistically stationary characteristics of a cultural group of subjects which have evolved over a long time horizon. Their response to specific social dilemma situations are then revealed through the present-day experiments.

In particular, when provided with the opportunity to punish norm deviators at own costs, altruistic behavior is manifested [25], [26], [55]–[57]. Even in one-shot interactions in public good games in which reputation and reciprocal effects are absent, costly punishment, which at a first sight seems to be in contradiction with individual fitness maximization, natural selection and rational choice theory, is frequently observed [25], [26], [58], [59]. One should, however, keep in mind that other patterns of behaviors may have emerged in the presence of different norms, environmental conditions and genetic endowments. E.g. subjects from 15 diverse populations display various behavioral patterns when playing an ultimatum game [24]. The diversity of behavioral traits found in different human cultures may result from different evolutionary trajectories as well as from distinct relative influences of the cultural versus genetic heritages and a varying intensity of the selection pressure [24], [49], [60], [61].

The co-evolutionary dynamics and inter-dependencies of genes and cultural norms constitute our starting point to understand the properties of our prosocial behavior and our sense of fairness, as observed in lab experiments, field studies and, of course, in real life. To identify and fully understand the mechanisms underlying our prosocial behavior, we design an evolutionary simulation model that mimics the dynamics of individuals being exposed to a social dilemma situation. To verify our theoretical results, we compare them with observations previously obtained in three independently conducted lab experiments. As a most important result, we find that evolution favors a build-in predisposition for fairness concerns: In the presence of a sufficiently large selection pressure, individuals inevitably develop an aversion to unfairness. Secondly, the dislike of unfair situations - not to be confused with a preference for fairness in general - promotes altruistic behavior in the form of costly punishment that occurs even in one-shot interactions as frequently observed in lab experiments. Thus, costly punishment is a consistent consequence of our conditional evolutionary predisposition to unfairness aversion.

In the following section, we will present our model, motivate, discuss and verify the obtained results and draw conclusions about the evolution of fairness preferences, altruism and moral behavior.

## Method

### 1 Design from public goods game experiments

We develop a simulation model consisting of synthetic agents that describes the long-term co-evolution of cultural norms and genes accounting for fairness preferences and costly punishment behavior in populations being exposed to a competitive voluntary contribution dilemma. Specifically, we compare our model with the results of three public goods game experiments conducted by Fehr/Gachter and Fudenberg/Pathak [25], [26], [59]. Even though the punishment of an agent B by agent A reduces the fitness of both and thus might be considered more as spiteful rather than an altruistic behavior, we use the term “altruistic” because the punishment of agent B by A increases in relative terms the fitness of other agents who participate in the same public goods game.

Our modeling strategy is to see the empirical observations in the experiments as a snapshot within a long-term evolutionary dynamics: on the short time scales of the experiments, the traits of the human players probed by the games can be considered fixed for each player. These traits might be encoded in the cultural context, in genes, or both.

Our model does not aim at simulating and explaining strategic short-term behavior of agents in social dilemmas, but instead mimics the culture-gene co-evolution that has occurred over tens of thousands of years. Aiming at two goals, we validate our model by comparing its results with the observed behavior in the experiments. In a first step, we quantitatively identify the underlying other-regarding preference relation that explains best the contemporary behavior. Here, we specifically look into a set of common assumptions made by researchers to account for fairness preferences and its observable consequences in the form of altruistic punishment behavior. Other-regarding preferences are expressed as inequality or inequity aversion. In our definition, inequality aversion refers to the dislike of unequal profits, ignoring a potential inequality in the individually contributed efforts. In contrast, inequity aversion relates the personal profits directly to the personal efforts that has been contributed to the group project. For instance, consider two agents A and B who contributes 70% and 30% respectively to the success of a project that pays 50 monetary units to each of them. If agent A is inequality averse, she will not feel uncomfortable or exploited by the equal sharing for the gains. In contrast, if she is inequity adverse, she will be unhappy to receive only half of the gains while having contributed more.

Initialized with different variants of these other-regarding preferences, the traits of our agents converge after long transients to statistically stable values, which are taken to describe the present-day characteristics of modern humans. In a second step, we verify that the identified preference relation which explains best the contemporary behavior is evolutionary stable and dominates the remaining variants of self- and other-regarding preferences. We do this by allowing the set of analyzed preferences to co-evolve over time within a heterogeneous population. In this way agents can assort, converge and establish an evolutionary stable other-regarding preference in their behavior. Our final goal is to reveal the ultimate mechanisms and the conditions under which agents develop spontaneously a propensity to “altruistically” punish, starting from an initial population of self-regarding and selfish-acting non-punishers.

The design of our model is inspired by three public goods game experiments with punishment conducted by Fehr/Gachter and Fudenberg/Pathak [25], [26], [59]. In these experiments, subjects - here undergraduate students from the Federal Institute of Technology (ETH) and the University of Zurich as well as subjects from the Boston area universities - are arranged in groups of 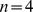 persons and play a two stage game. At the beginning of each period, in stage one, subjects received an initial endowment of 20 monetary units (MUs). Thereafter, subjects could invest 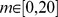 MUs to a common group project, which returned 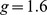 MUs for each invested MU. The total return from the project was equally split and redistributed to all group members. Thus, the return per capita was 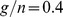. As long as 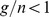, the game has a vivid social dilemma component, since it is rationally optimal not to cooperate, even though the group is better off if each member cooperates: if all agents contribute one MU (cooperate), they each obtain 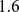 MU. If only one does, the three others (free-riders) pocket 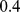 MU on top of their own uninvested MU while the single contributor is left with just 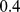 MU and thus takes a loss of 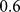 MU. Thus the setup is susceptible to defection through material self-interest and we consider the subjects' investment as their level of cooperation.

In the second stage of the game, subjects were provided with the opportunity to punish other group members, after they had been informed about the individual contributions. In [59], subjects also played an unobserved treatment in which they learned the contributions of other group members not until the last period has been played. However, this variation in the design of the experiment did not lead to a significantly different level of observed punishment. The use of punishment was associated with costs for both parties, in which each MU spent by a punisher led to 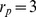 MUs taken from the punished subject [26], [59]. In [25], the punisher paid approximately 2 MUs to take an additional 10% from the punished subject's period profit. Experiments were played both in a partner treatment [25], in which the group composition did not change across periods, and in a stranger treatment [25], [26], [59]. In the later, subjects were reassigned to new groups at each period using an anonymous random matching procedure and thus were only engaged in one-shot interactions during the entire runtime of the experiment. In total, the experiments were played for 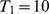 [25], [59] and 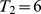 periods [26] respectively.

The data from Fehr/Gaechter and Fudenberg/Pathak as well as from several other public goods experiments [55]–[57] show that people, if provided the opportunity, frequently punish defectors, even if this is costly to themselves and not immediately observable to others. We should mention that different patterns of behavior may have emerged in different cultural areas. We address this point below in the computational model and in the section concerning the model assumptions.

In the case of repeated interactions, as in the partner treatment, such behavior might be explained by the “direct reciprocity” mechanism. What is more surprising is that subjects continue to punish at a cost to themselves even in one-shot interactions for which there is no feedback mechanism in action that would work e.g. by direct or indirect reciprocity. This costly punishment behavior is often referred to as “altruistic” to emphasize the conflict with the behavior expected from purely rational agents. The question we address here is why humans behave in a way that seemingly contradicts individual fitness maximization and rational choice.

### 2 Computational model and assumptions

We construct an evolutionary simulation model adapted from the design of the experiments in [25], [26], [59] that consists of a population of agents who play a public goods game with punishment. In this model, agents are characterized by three traits. The first two traits characterize the agent's level of cooperation 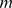 and their propensity to punish 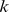. The third trait 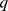 characterizes the agent's preferences for self- and other-regarding behavior, respectively. All traits can adapt and evolve over long periods according to generic evolutionary dynamics: individual learning and population adaptations by selection, crossover and mutation. In this context we define these dynamics by:

- individual learning: the changing of behavior during the lifetime of an agent, e.g. through learning.
- selection: the evolutionary selection of individuals based on their fitness.
- cross-over: the recombination of genes/traits of two or multiple agents during the reproduction process.
- mutation: the random alteration of individual genes/traits during the reproduction process.

In order to capture the possible evolution of the population, agents adapt and die when unfit. Newborn agents replace dead ones, with traits taken from the pool of the other surviving agents. The learning and adaptation/replication dynamics are described in detail in section 3 and 4, respectively.

**Figure 1:**
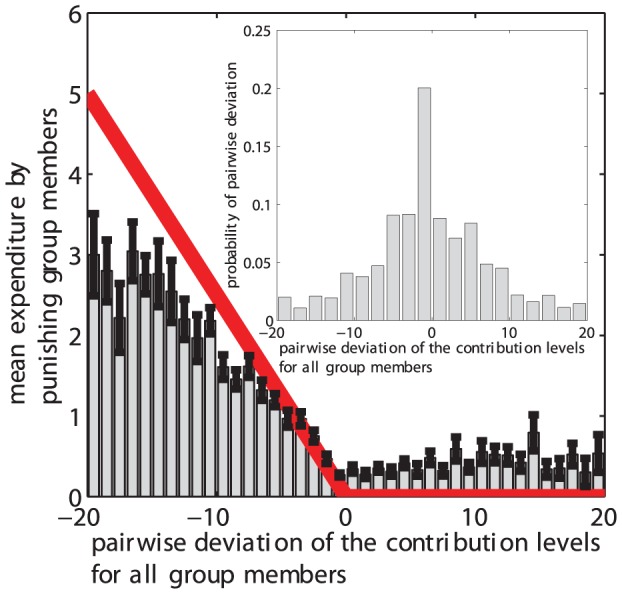
Mean expenditure of a given punishing member as a function of the deviation between her contribution minus that of the punished member, for all pairs of subjects within a group, as reported empirically [25], [26], [59]. The error bars indicate 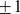 standard error around the mean. The straight line crossing zero shows the average decision rule for punishment that our agents spontaneously evolve to at long times. Its slope 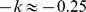 defines the average propensity 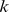 to punish (see the main text). The anomalous punishment of cooperators, corresponding to the positive range along the horizontal axis, is not considered in our model. The inset shows the relative frequency of the pairwise deviations.

A given simulation period 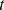 is decomposed into two sub-periods:

1. **Cooperation:** Each agent 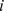 chooses an amount of 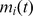 MUs to contribute to the group project in period 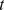. This value of 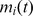 reflects the agent's intrinsic willingness to cooperate and thus is referred to as her level of cooperation. As in the experiments, each MU invested in the group project returns 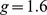 MUs to the group. Combining all the contributions by all group members and splitting it equally leads to a per capita return given by equation (1).
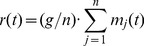
(1)This results in a first-stage profit-and-loss (P&L) of
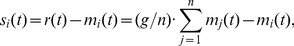
(2)for a given agent 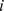, which is equal to the difference between the project return and its contribution in period 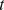. The willingness to cooperate embodied in trait 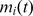 evolves over time as a result of the experienced success and failures of agent 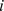 in period 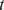. The learning and adaptation/replication rules are described in detail in sections 3 and 4.
2. **Punishment:** Given the return from the group project 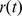 and the individual contributions of the agents, 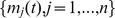, which are revealed to all, each agent may choose to punish other group members according to the rule defined by the equation (3) below. To choose the agents' decision rules on when and how much to punish, we are guided by figure 1. Resulting from the data of three experiments, figure 1 shows the empirically reported average expenditure that a punisher incurs as a reaction to the negative or positive deviation of the punished opponent.

One can observe an approximate proportionality between the amount spent for punishing the lesser contributing agent by the greater contributing agent and the pairwise difference 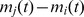 of their contributions. The figure includes data from all three experiments [25], . In our model, this linear dependency, with threshold, is chosen to represent how an agent 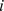 decides to punish another agent 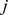 by spending an amount given by
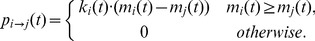
(3)


This essentially corresponds to punishment being directed only towards free riders. We assume a linear dependency between 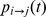 and 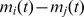, because it can frequently be observed in experiments conducted in the western cultural area. Other patterns of behavior, in particular those representing spiteful behavior such as antisocial punishment, may be dominant in other cultural areas [28], [62]. However, we do not account for different punishment behaviors and thus cannot generalize our model with respect to distinct cultural identities and the associated behaviors. The coefficient 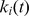, which represents the propensity to punish, is the second trait that characterizes agent 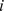 at time 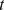. It is allowed to vary from agent to agent and it evolves as a function of the successes and failures experienced by each agent, as explained in sections 3 and 4. Given that certain other-regarding preferences are active, we will show that evolution makes the punishment propensities 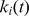 self-organize towards a value fitting remarkably well the empirical data, without the need for any adjustment.

As a result of being punished, the fitness of the punished agent 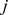 is reduced by the amount spent by agent 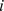 multiplied by the punishment efficiency factor 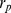. As in the experiments, we fix the punishment efficiency factor to 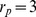. In the first experiment of Fehr/Gachter [25], the punishment efficiency factor was determined based on the first stage payoff of the punished individual. However, it can be considered to be approximately equal to the factor 3 as in the remaining two experiments.

The total P&L 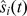 of an agent 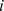 over one period of her lifetime is thus the sum of three components: (i) her first stage P&L 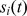 from the group project (equation (2)), (ii) the MUs 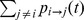 spent to punish others and (iii) the punishments of MUs 
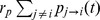
 received from others, where 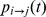 and 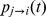 are given by (3):
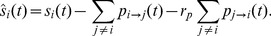
(4)
Equation 4 represents the second stage P&L of agent 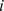 in period 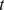.

### 3 Behavioral learning dynamics

It has been argued [63]–[66] that humans (and our ancestors) are likely to use heuristics and inductive reasoning to make decisions. In particular, this means that humans tend to replace working hypotheses with new ones when the old ones cease to work. We adopt this bounded rational approach to define the adaptation mechanism that controls the dynamics of the propensity to punish and the level of cooperation.

The first two traits 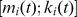, characterizing each agent 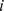 at a given period 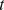, evolve with time according to standard evolutionary dynamics: adaptation, selection, crossover and mutation. While selection, crossover and mutation operate on the individual fitness level, i.e. are controlled by the birth-death process, adaptations are individually performed by each agent during its lifetime. We model this phenotypic expression that controls the adaptation dynamics using a third trait, 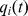. In particular, we focus on the set of inequality and inequity aversion preferences, which have been identified as important determinants in the human decision process and that of other species [1], [40], [67]–[71].

The following six preference types represent the fundamental set of variants of inequality and inequity aversion preferences: (A) inequity averse, (B) inequality averse, (C) disadvantageous inequity averse, (D) advantageous inequity averse, (E) disadvantageous inequality averse and (F) advantageous inequality averse. “Disadvantageous” indicates that agents are only inequality/inequity averse if the inequality/inequity plays to their disadvantage, while “advantageous averse” agents do the opposite. In contrast, pure inequality or inequity averse agents dislike both situations in which they are discriminated against or are discriminating others. We also analyze purely self-regarding and selfish-acting agents (G), i.e. agents who adapt their traits independently of the actions and the outcomes of other agents.

**Figure 2:**
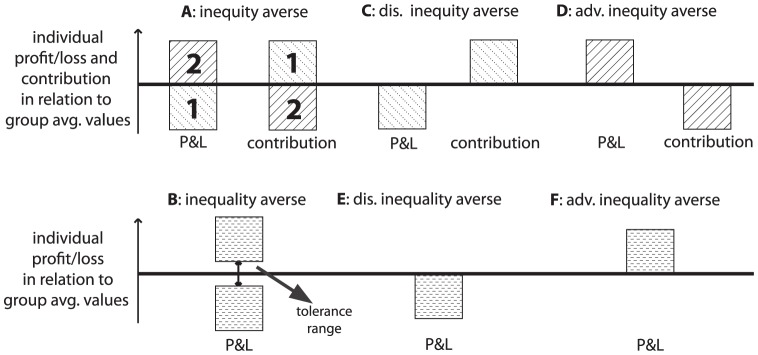
Scheme of the different possible variants of inequality and inequity aversion preferences introduced in the text.

Figure 2 depicts schematically the possible variants of inequality and inequity aversion preferences introduced above. While inequity aversion (first row) is determined by a combinatorial condition relating the P&L to the performed effort (contribution) of an agent, inequality aversion (second row) is determined only by the agent's P&L value. Disliked regions of individual P&Ls (inequality aversion) or combinations of P&Ls and contributions (inequity aversion) are highlighted by boxes filled using the same pattern: E.g. inequity averse agents (first row, left column) dislike situations in which they contribute more than the average and their P&L is less than the average (combination indicated by 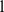) or vice versa (combination indicated by 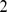).

In contrast to 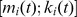, which are continuous measures, 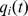 represents a discrete indicator variable that corresponds to a specific boolean expression. The associated boolean expression translates into a specific adaptation condition that expresses a self- or other-regarding preference relation. If a particular condition becomes satisfied, an unbiased adaptation of 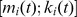 is triggered. This allows each agent to adapt 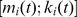, either solely based on the individually experienced P&L values, or depending on the P&L and contributions of all group members. Our model implementation allows us to pairwise compare different self- and other-regarding preferences, i.e. a heterogeneous population can co-evolve along two different adaptation rules 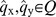 across time. The value 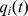 determines which of the two conditions 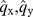 is active for agent 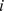: if agent 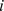
's indicator value 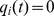, then she adapts 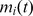 and 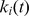 according to rule 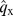. In contrast, if 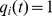, adaptation occurs according to the second rule 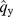.

The following list describes the set of analyzed phenotypic expression 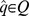 in detail:

 A: **Inequity averse agents**: such an agent 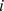 updates her cooperation level and her propensity to punish according to eq. (5) below, if…

 …she has contributed less than (or equally) to her group fellows (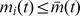), where the average 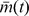 is performed over the contributions of the other members of her group and, at the same time, has received a total P&L 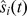 defined in (4) larger than (or equal) to the group average (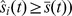, where the average 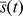 is performed over the other group members…

 …or she has contributed more than (or equally) to her group fellows (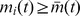) and, at the same time, has received a total P&L less than (or equal) to the group average (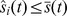).

 For inequity averse agents, the boolean expression is defined as 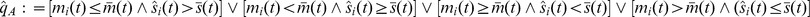.

 B: **inequality averse agents**: such an agent 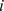 updates her cooperation level and her propensity to punish if her P&L 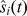 given by (4) is not within a specific tolerance range 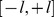 around the average P&L of the other members of her group, i.e. if 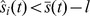 or 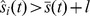. When this occurs, agent 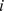 updates her traits 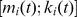 according to equation (5). It is clear that inequality averse agents do not take the individually contributed efforts explicitly into account, in contrast with the inequity aversion agents (A).

 For inequality averse agents, the boolean expression reads 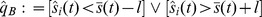


 We run multiple simulations initialized by different values for 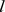 as presented in the results section.

 C: **disadvantageous inequity averse agents**: as for agents of type (A), disadvantageous inequity averse agents compare their P&L to their contributions, however they only dislike situations in which the inequity is detrimental to them. If an agent 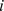 has contributed equally or more than her fellows in the group (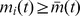) and, at the same time, has received a total P&L 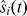 defined in (4) smaller than or equal to the group average (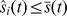), then she updates her traits 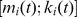 according to eq. (5).

 For disadvantageous inequity averse agents, the boolean expression is defined by 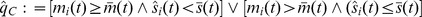


 D: **advantageous inequity averse agents**: these agents correspond to the antithesis of agents of type (C). If an agent 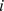 has contributed equally or less than her fellows in the group (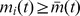) and, at the same time, has received a total P&L 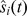 defined in (4) larger than or equal to the group average (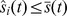), then she updates her traits 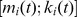 according to eq. (5).

 For advantageous inequity averse agents, the boolean expression is 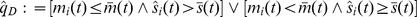


 E: **disadvantageous inequality averse agents**: these agents only dislike situations in which the inequality is to their disadvantage. An agent 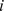 updates her cooperation and her propensity to punish only if her P&L 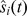 given by (4) is smaller than the average P&L of the other members of her group, i.e. 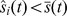. When this occurs for an agent 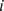, she updates her traits according to equation (5).

 The corresponding boolean expression for disadvantageous inequality averse agents is 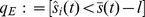


 F: **advantageous inequality averse agents**: these agents only dislike situations in which the inequality is to their advantage as opposed to setup (E). An agent 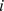 updates her cooperation and her propensity to punish only if her P&L 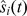 given by (4) is larger than the average P&L of the other members of her group, i.e. 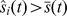. When this occurs for an agent 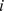, she updates her traits according to equation (5).

 Advantageous inequality aversion is defined by the boolean expression 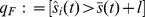


 G: **self-regarding agents**: such an agent updates her cooperation and propensity to punish if her P&L 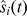 given by (4) turns out to be smaller than the P&L in the previous period 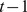.

 Pure self-regarding and selfish behavior is defined by the boolean expression 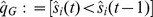


In addition, each agent needs at least to consume an amount of 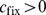 per period in order to match the minimum costs of living, i.e. this value reflects the absolute lower limit required for survival. Thus agents in all dynamics (A–F) additionally adapt their traits if their P&L is less than 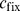 in avoidance of becoming extinct.

The update an agent performs if the predominant condition from the set of conditions 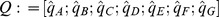 applies consists in an unbiased random increment according to
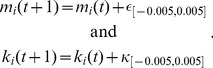
(5)


The random variables 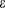 and 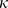 are uniformly distributed within the interval indicated in the subscript. Since contributions and punishment expenditures are non-negative, draws of 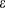 and 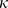 are truncated to avoid realizations that would lead to negative values of 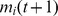 and/or 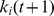. Our results are robust to changes of the width of the interval, as long as it remains symmetric around zero.

### 4 Adaptation and Replicator Dynamics: Selection, crossover and mutation

In addition to the learning dynamics of the agents' traits 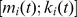 described above, adaptation in the form of survival and fertility selection occurs on a population level by replacing under-performing agents.

Adaptation is a process that affects the individual's fitness as a result of facing short- and long-term changes in the environment [72]–[74]. In the context of our model, adaptation translates into a three-stage process: selection, cross-over and mutation. As we do not include a population dynamic, our model assumes a constant group size equal to 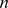, with each death being followed by a corresponding birth. We tested our model with the following three variants of the selection mechanisms:

 *S1*: In the first variant, consumption absorbs an amount 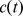 of the agents' fitness at period 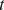. The consumption for each agent is defined proportional to the average P&L of the group but at least requires to meet a minimum threshold of 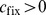 per period in order to satisfy a minimum survival capability as described before. Thus the consumption in period 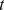 is determined by:
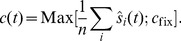
(6)This setup represents a realistic driving force to select for successful traits, i.e. those traits carried by agents that perform better than the group average over time. Selection occurs if an agent's wealth drops below zero, i.e. 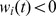. In this case, the agent dies and is replaced.

 *S2*: In the second variant, the death- and rebirth-event of an agent occurs with a probability proportional to the wealth of the agents: For each simulation period, the agent with the lowest wealth (fitness) in the group dies with a probability of 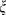 and is subsequently replaced. We have varied 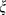 in a range 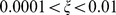 resulting in essentially the same output. To avoid negative values of wealth, which might occur as a result of continuously realized negative P&L values, agents are endowed with an initial wealth 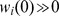.

 *S3*: In the third investigated variant, selection occurs based on a simple mechanism with non-overlapping generations, i.e. all agents have the same predefined lifespan. After one generation has reached its maximum age, the entire population of agents is replaced. Agents receive an initial endowment with 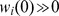 to prevent negative values of wealth (fitness) during their lifetime.

Our results are robust to all three selection mechanisms (*S1*, *S2* and *S3*), i.e. all variants essentially create the same quantitative output. To be specific, without loss of generality, we obtained all results described in the following sections using selection dynamic *S1*.

To simulate fertility selection and variation by cross-over, we initialize reborn agents with traits 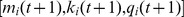 that are inherited from the surviving agents with a probability proportional to their fitness, respectively proportional to the agents in the previous generation in case of *S3*. This simulates, that successful individuals produce more offsprings, by propagating more successful traits more strongly than less successful ones and ensures variation by a mixing of the trait/gene pool. Finally, we add mutation in form of a small random noise to the inherited traits. In detail, the process of crossover and mutation for the first two traits, 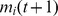 and 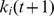, is determined as follows:
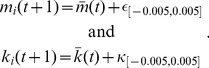
(7)

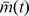
 and 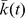 correspond to the fitness weighted average values calculated over the surviving (*S3*: previous) population and 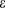 and 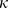 reflect the individual mutation rates in the form of an unbiased uniformly distributed random increment over the interval indicated by the subscript. Again, draws of 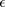 and 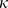 are adjusted in a way to ensure the non-negativeness of the 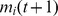 and 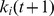 values.

**Figure 3:**
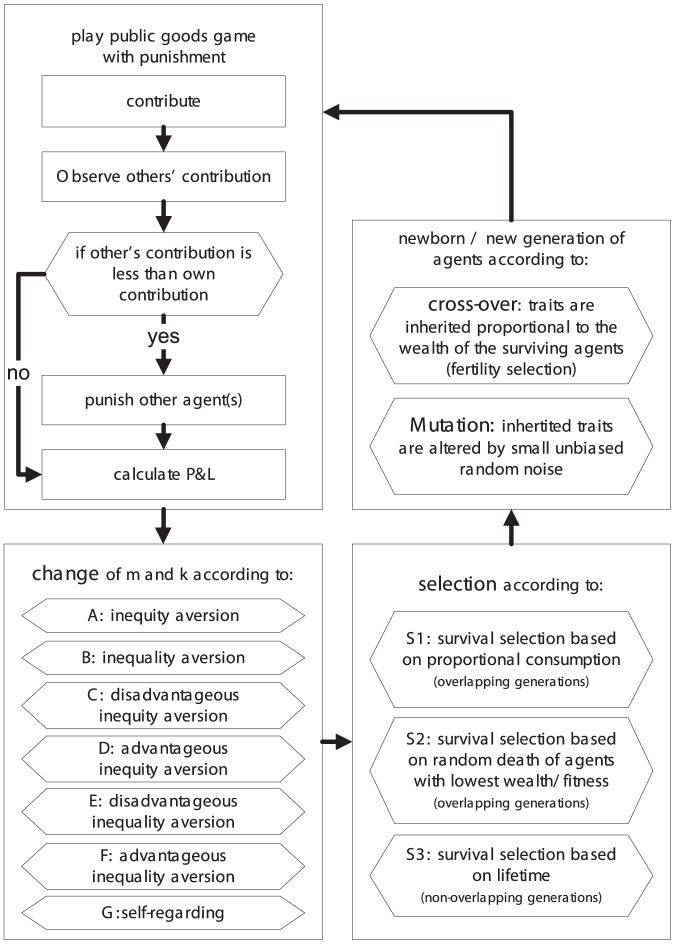
Schematic depiction of the evolutionary model flow including *adaptation*, *selection*, *cross-over* and *mutation*.

Crossover and mutation for the discrete indicator variable 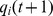 occurs analogously as follows:
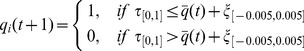
(8)First, the fitness weighted average of the surviving (*S3*: previous) population 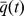 is calculated and mutated by a random variable 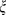 that is uniformly distributed in 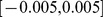. Second, a 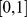-uniformly distributed random number 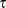 is drawn and compared to the value 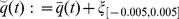. If 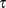 is less than or equal to 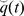, 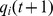 becomes one and zero otherwise. Figure 3 summarizes and outlines the model flow schematically.

In a nutshell, our model is essentially based on the following assumptions:

- Agents play a public goods game with punishment opportunity.
- Agents can only punish other agents who contributed less than themselves (free-riders), i.e. we do not consider spiteful behavior of agents.
- The model is intended to mimic the long-term gene-culture co-evolutionary dynamics: We do not include strategic short-term behavior in the agents' behavior, i.e. agents do not have a memory about the others' behavior in previous periods.
- Agents are characterized by three traits which are updated based on standard evolutionary dynamics.
- Evolutionary updates of traits are marginal and only controlled by the current active other or self-regarding preference relation.
- An agent's fitness is defined relative to other group members: agents who outperform others over time spread their traits with a higher weight than less successful agents.
- Agents need to consume a certain amount of their fitness per period, which is tied to the average payoff from the public good. We also tested other variants in section 0.4 (S1–S3) with essentially the same results.

## Results

This section is structured in two parts. In the first part, we aim at determining which superordinate regime 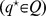 of self- or other-regarding preferences might have led our ancestors to develop traits promoting costly or even altruistic punishment behavior to a level that is observed in the experiments [1], [75]. To answer this question, we let the first two traits 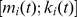 co-evolve over time while keeping the third one, 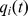, fixed to one of the phenotypic traits defined in 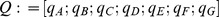. In other words, we account only for a homogeneous population of agents that acts according to one specific self-/other-regarding behavior during each simulation run. Starting from an initial population of agents which displays no propensity to punish defectors, we will find the emergence of long-term stationary populations whose traits are interpreted to represent those probed by contemporary experiments, such as those of Fehr/Gachter or Fudenberg/Pathak.

The second part focuses on the co-evolutionary dynamics of different self- and other-regarding preferences embodied in the various conditions of the set 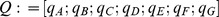. In particular, we are interested in identifying which variant 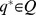 is a dominant and robust trait in presence of a social dilemma situation under evolutionary selection pressure. To do so, we analyze the evolutionary dynamics by letting all three traits of an agent, i.e. 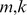 and 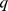 co-evolve over time. Due to the design of our model, we always compare the co-evolutionary dynamics of two self- or other-regarding preferences pairwise, and we consider all possible combination in 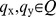 with 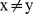. Again starting from an initial population of agents with no disposition for other-regarding behavior and for altruistic punishment, we report below a remarkable consistency between (a) the evolutionary dominance of a variant of other-regarding behavior and (b) our findings from the first part of the analysis that focused on the empirical identification and validation.

The results presented below correspond to groups of 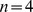 agents with a punishment efficiency factor of 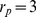 and a per capita return per contributed MU of 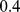 (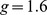) as in the experiments. The minimum consumption value has been set to 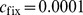. We have run our simulation with thousands of independent groups over 10 million simulation periods.

### 5 The effect of other-regarding preferences on the evolution of altruistic punishment

To identify if some, and if so which, variant of self- or other-regarding preferences drives the propensity to punish to the level observed in the experiments, we test each single adaptation conditions defined in 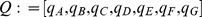. In each given simulation, we use only homogeneous populations, that is, we group only agents of the same type and thus fix 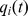 to one specific phenotypic trait 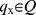. In this setup, the characteristics of each agent 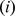 thus evolve based on only two traits 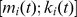, her level of cooperation and her propensity to punish, that are subjected to evolutionary forces.

Each simulation has been initialized with all agents being uncooperative non-punishers, i.e., 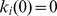 and 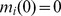 for all 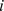
's. At the beginning of the simulation (time 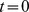), each agent starts with 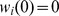 MUs, which represents its fitness. After a long transient, we observe that the median value of the group's propensity to punish 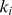 evolves to different stationary levels or exhibit non-stationary behaviors, depending on which adaptation condition (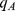,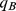,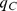,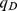,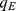,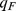 or 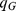) is active. We take the median of the individual group member values as a proxy representing the common converged behavior characterizing the population, as it is more robust to outliers than the mean value and reflects better the central tendency, i.e. the common behavior of a population of agents.

**Figure 4:**
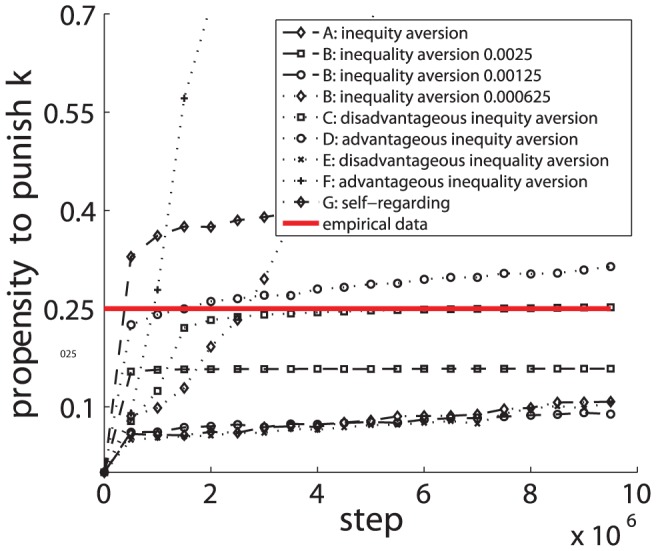
Evolution of the propensity to punish as a function of time. The values correspond to the population's median of the individual 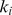 values as a function of time for the seven different adaptation dynamics (A to G). The values for each adaptation dynamic result from 800 system realizations with a total of 3200 agents. The empirical median value calculated from all three experiments of Fehr/Gachter's and Fudenberg/Pathak [25], [26], [59] is shown as the continuous horizontal line. For adaptation dynamic (B), the plot shows the obtained median values for all tolerance range parameters 
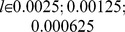
. The parameters of our simulation are: 
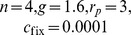
.

**Figure 5:**
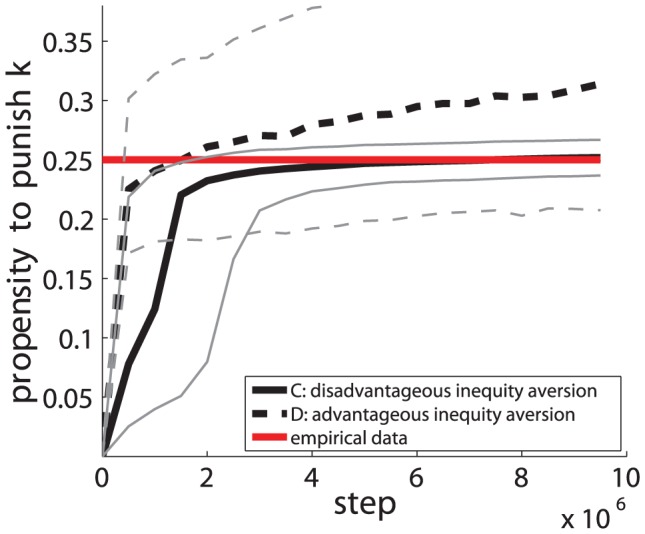
Magnification of figure 4 for adaptation dynamics C and D including their 20/80 quantiles (thin continuous grey line (C) and thin dotted grey line (D)). The horizontal continuous line corresponds to the median value of the empirically observed propensities to punish.

Figure 4 compares the evolution of the median of the propensities to punish obtained from our simulation for the six adaptation dynamics (A to F) with the median value calculated from the Fehr/Gachter's and Fudenberg/Pathak empirical data [25], [26], [59]. The propensities to punish in the experiment have been inferred as follows. Knowing the contributions 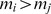 of two subjects 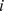 and 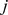 and the punishment level 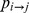 of subject 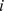 on subject 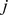, the propensity to punish characterizing subject 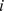 is determined by
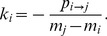
(9)Applying this recipe to all pairs of subjects in a given group, we obtain a measure of propensities to punish per group. Sampling all groups and all periods, we calculate the median of all 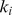 values as shown in figure 4 (continuous horizontal line). Figure 5 additionally shows a magnification of figure 4 for adaptation dynamics C and D including their 20/80 quantiles.

Figures 4 and 5 reveal that purely self-regarding and selfish-acting agents adapting their traits according to dynamics (G) remain weak-punishers as shown in figure 4. In contrast, for agents endowed with inequality or inequity aversion preferences (adaptation conditions A to F), different stationary and non-converging states of the propensity to punish emerge spontaneously, each with different characteristics.

**Result 1**: *For all adaptation rules (A to F), it holds that altruistic punishment has emerged endogenously as a trait in a competitive social dilemma scenario that is subject to evolutionary selection pressure.*

In detail, we find that, for self-regarding and selfish-acting agents (dynamics G), the level of punishment that evolved remains too small to explain the empirical results of Fehr/Gachter and Fudenberg/Pathak. For the inequality averse population (B), we find that, for a set of reasonable values of the tolerance range parameter 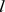, the empirical distribution can not be reproduced. Figure 4 shows the median value of the propensity to punish for adaptation dynamics B with the following values of the tolerance range parameter 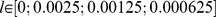. While a large tolerance range causes altruistic punishment to remain weak, a narrow tolerance range results in continuously increasing and thus non-stationary levels of punishment. For inequity- and altruistic inequity averse agents (dynamics A and D) as well as for disadvantageous inequality- and altruistic inequality averse agents (dynamics E and F), we find levels of altruistic punishments that far exceed the empirical evidence. We find that the adaptation dynamics C (disadvantageous inequity averse agents) causes the values 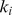 of the propensity to punish to converge towards the empirically observed norm. The quantitative comparison with the Fehr/Gachter and Fudenberg/Pathak experiments supports the hypothesis that human subjects are well-described as being disadvantageous inequity averse (dynamics C), corroborating and complementing previous evidence [42], [43], [45]. A Mann-Whitney test does not reject the equality of the median values between the results obtained by the adaptation dynamics C and the empirical data observed in the experiments with a p-value of 0.943. For all other adaptation dynamics, the equality of the obtained median propensity to punish and the experimental value is clearly rejected. Also, the 95% confidence interval for the sample median, either for each of the three experiment data sets independently, in pairs or as a whole does not allow us to reject adaptation dynamic C, whereas all other dynamics (A,B,D–G) can be rejected. The propensities 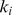 to punish exhibits a median around 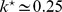, which means that most punishers spend an amount approximately equal to one-fourth of the experienced differences in contributions in the given setup with 4 players. Note that the value of the median around 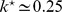 is close to the slope of the straight line fitting the empirical data shown in figure 1. This value 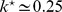 has also been identified analytically as a evolutionary stable strategy resulting from the maximization of an expected utility problem with disadvantageous inequity aversion preferences under evolutionary dynamics [76]. Given the simplicity of our model and of its underlying assumptions, it is striking to find such detailed quantitative agreement for one of our dynamics. This immediately raises the question of the generating underlying mechanisms that control these dynamics.

It is important to stress that the competitive evolutionary environment with its distinct selection pressure has no build-in mechanism that ex ante favors the emergence of altruistic behavior such as the costly punishment of defectors. Rather, the interplay of the evolutionary selection- and the individual adaptation-processes causes the propensity to punish 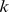 to evolve to a level that matches the empirical observations. Remarkably, a symmetric inequity aversion, i.e. an aversion for disadvantageous and advantageous inequity, is not needed as a condition to let altruistic punishment emerge.

**Result 2**: *A purely disadvantageous inequity aversion is sufficient to explain the spontaneous emergence of altruistic punishment, with a median level of the propensity to punish that precisely match empirical data.*

**Figure 6:**
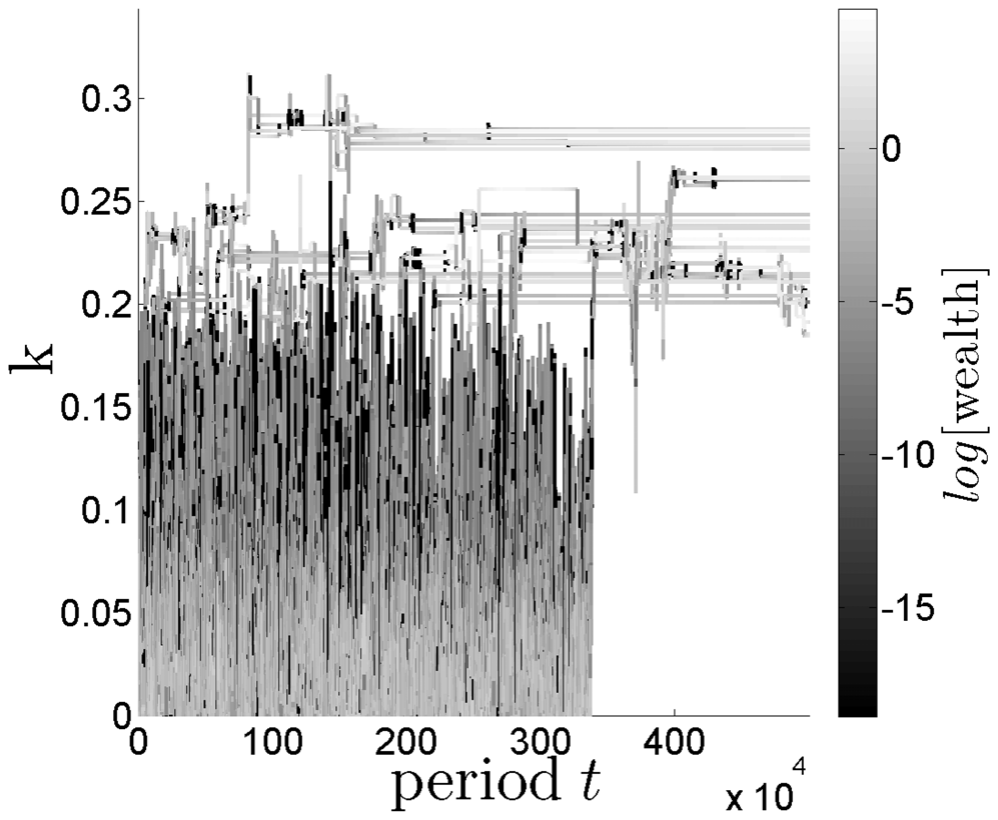
Evolution of the propensity to punish 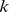 (y-axis) over 5 million time steps (x-axis) (sample taken every 100 steps) resulting from 8 system realizations with a total of 32 agents in 8 groups. The shade of grey indicates the evolution of the agents' fitness values.

In order to understand how altruistic traits are selected in our simulation model, we analyze the evolution of the individual realized fitness- and P&L-values across time. Additionally, we inspect the micro behavior of the adaptation conditions A–G on a per step level to understand why and when agents adapt their traits 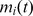 and 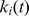. Figure 6 shows the evolution of a population of disadvantageous inequity averse agents (adaptation dynamics C). The figure reveals that the preference for disadvantageous inequity aversion together with the evolutionary dynamics, in form of survival and fertility selection, is responsible for the emergence of altruistic punishment behavior in our model:

Figure 6 shows the average group fitness of the agents across time on a logarithmic scale. We use a logarithmic scale as it better highlights the wealth dynamics across time. This plot reveals the existence of two evolutionary attraction points 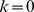 and 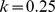, which are identified by two discrete horizontal ranges around 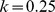 and 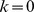 for which the fitness takes the largest values (brighter shape of grey). Both evolutionary equilibria are separated by a range of values 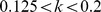, in which the evolution is unstable (darker grey shape). Supporting figures for this effect are presented in the supporting information section.

As described above, fertility selection occurs by replacing dead agents with newborns whose traits are taken proportional to the wealth of the surviving group members. This results in 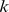-values that are associated with a higher fitness to dominate and to spread in the population as a function of time in the presence of an ongoing deaths/births process. Figure 6 shows that more and more agents with 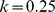 start to dominate the heredity transmission mechanisms, i.e. they spread their propensity to punish (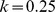) much more than those with 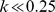. This is because their fitness is higher and, at the same time, the deaths of agents with 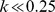 occur more frequently. This becomes visible in figure 6 in the form of an increasing brighter shape of grey along the time line for realizations corresponding to a 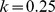, while those with 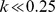 remain at a lower fitness level and disappear by-and-by.

In summary, we observe the co-evolution of three processes.

1. Aversion to disadvantageous inequity makes agents adapt their behavior and explore values of their propensity to punish at levels 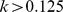.
2. This leads them into a evolutionary unstable state associated with the range 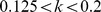.
3. Subsequently, the evolutionary dynamics in the form of selection, cross-over and mutation, makes agents converge towards an equilibrium of their propensity to punish at a value around 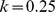.

This equilibrium emerges as a result of the aversion to disadvantageous inequitable outcomes in combination with the evolutionary survival condition P&L-consumption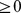. These two conditions can only be fulfilled simultaneously for 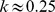.

**Figure 7:**
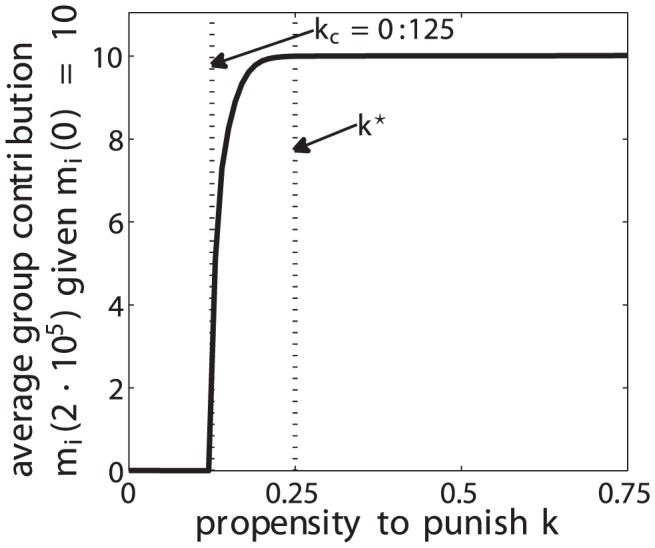
Average group contribution for a group of 4 agents as a function of 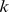 for dynamic C (disadvantageous inequity aversion) after an equilibrium time of 20,000 simulation periods and for 1000 system realizations. 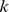
 is fixed to the corresponding value on the x-axis and the initial contribution 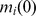 for all agents 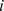 of a group is randomly drawn form a uniform distribution in 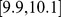.

**Figure 8:**
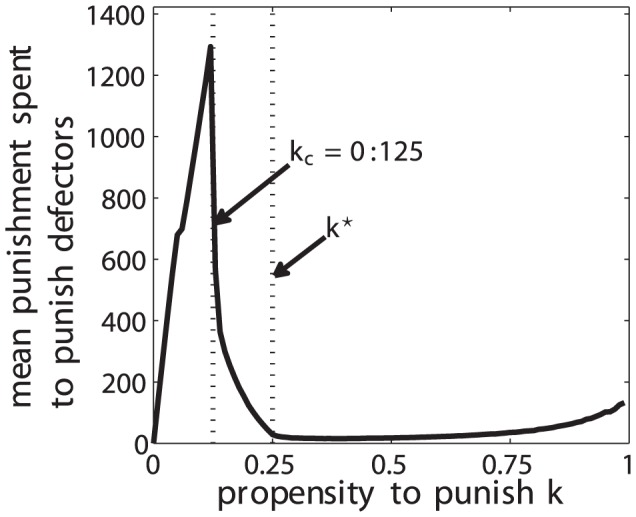
Average punishment spent to punish defectors for a group of 4 agents as a function of 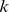 after an equilibrium time of 5,000,000 simulation periods and for 3200 system realizations. 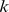
 is fixed to the corresponding value on the x-axis and the initial contribution 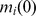 in period 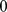 for all agents 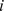 of a group is randomly drawn form a uniform distribution in 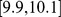. A value of 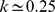 corresponds to an optimal value of the propensity to punish associated to a minimum of the global punishment expenditure.

We further explore and analyze the sensitivities of a population of agents with respect to the propensity to punish 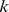. This allows us to substantiate the existence of an evolutionary stable equilibrium at 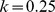. First, we analyze the sensitivity of the level of cooperation 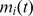 for fixed values of 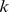, ranging from zero (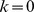) up to excessive punishment behavior with 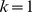. Figure 7 shows the average level of cooperation in a group of 4 agents after a transient period of 20,000 simulation periods for 1000 system realizations as a function of the propensity to punish 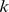. The level of cooperation for all agents was initialized by a value drawn from a uniformly distributed random variable in 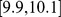. This figure reveals that the level of cooperation undergoes a phase transition at the critical value 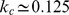, at which it becomes non-zero and grows rapidly to a saturation value. For propensities to punish larger than 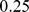, the level of cooperation remains constant at its saturation value. The value 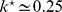 seems to be the minimum propensity to punish that enforces to sustain a maximum level of cooperation. This suggests that agents with a disadvantageous inequity aversion select an “optimal” propensity to altruistically punish defectors in to sustain cooperation in a group. To further substantiate this hypothesis, we interpret the intrinsic propensity to punish 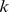 as a measure of deterrence. Figure 8 plots the average amount of MUs spent to punish a defector during 5,000,000 simulation periods for 3200 system realizations as a function of the propensity to punish 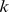. As in the setup of figure 7, the level of cooperation 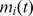 for all agents is initialized at period 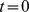 by a random variable uniformly distributed in 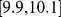. The results show clearly that for values of 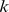 above the critical value of 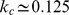, which corresponds to a higher level of deterrence, effectively less exertion of costly punishment is caused in order to maintain a certain level of cooperation. This responsive behavior was manifested in many empirical observations [77]–[80]. The value 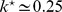 corresponds to the minimum overall punishment cost with a stable maximum cooperation level. This substantiates that disadvantageous inequity averse agents may have selected an “optimal” propensity to punish to sustain cooperation and prevent defection. Comparable results were obtained using a different simulation model, as reported in [81].

Figure 4 has shown that altruistic punishment emerges not only in the presence of disadvantageous inequity aversion but also in the presence of the other variants of other-regarding preferences (dynamics A,B, D–F). However, populations of agents initialized with dynamics A,B, D–F do not converge to evolutionary stable states. This means there exits no evolutionary dynamic with a statistically stationary behavior. A more detailed discussion is presented in the supporting information.

To give a rough idea about the evolutionary dynamics, we find that agents have an average lifetime of 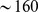 periods with a median value of 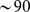 periods. Therefore, a typical simulation run allows the occurrence of tens of thousands generations.

### 6 The co-evolution of self- and other-regarding preferences

The results obtained in the previous section suggest that the punishment behavior of subjects observed in the experiments is driven by an aversion against disadvantageous inequity. Consequently, this raises the question whether the identified adaptation dynamic 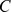 (disadvantageous inequity aversion) is an evolutionary stable and dominant trait that emerges and prevails in an competitive resource-limited environment in the presence of other variants of self- and other-regarding preferences. This can be verified by allowing agents with an aversion against disadvantageous inequitable outcomes to co-evolve along with other agents that act based on one of the remaining adaptation conditions (A,B,D–G) in our model.

In the following, we run our model with a population that consists of members who are either disadvantageous inequity averse or have the trait of one of the other self- or other-regarding preferences. In this way, we can compare the reciprocal effects of the co-evolutionary dynamics from each variant A,B,D–G against disadvantageous inequity aversion preferences. This results in 6 pairwise comparisons. In the beginning of each simulation run, the model is initialized with a preliminary homogeneous population of agents which are *not* disadvantageous inequity averse but moreover act according to one of the dynamics defined by A,B,D–G. The evolutionary updates of the two competing adaptation traits is performed as described in section 0.4, i.e. 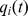 alternates between 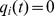 and 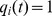 according to the results of selection, crossover and mutation.

**Figure 9:**
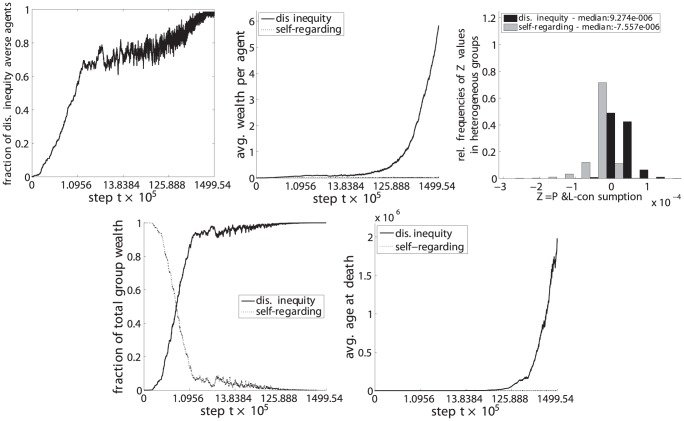
Disadvantageous inequity aversion (C) vs. self-regarding (G).

**Figure 10:**
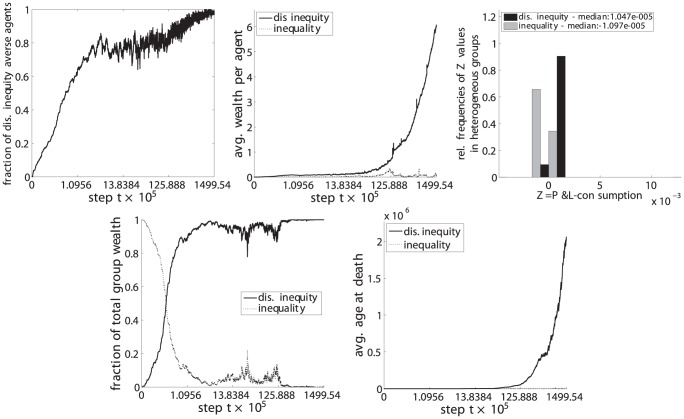
Dis. inequity aversion (C) vs. inequality aversion (B). Upper left: fraction of disadvantageous inequity averse agents in the population. Top center: average wealth per agent. Upper right: distribution of 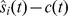 values for steps 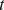 with heterogeneous groups. Lower left: fraction of the total population wealth. Lower right: average age of agents at death.

**Figure 11:**
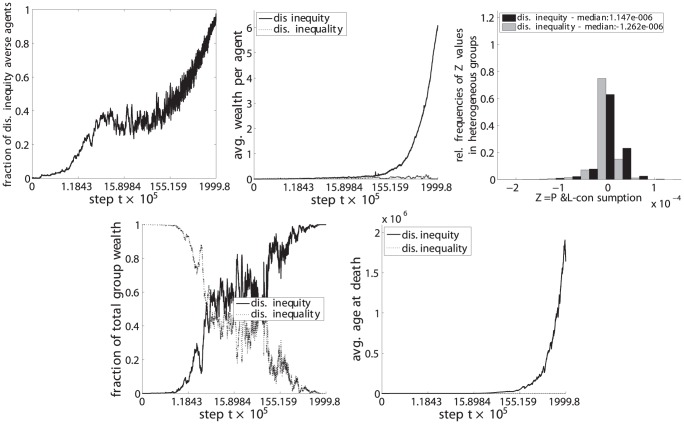
Dis. inequity aversion (C) vs. dis. inequality aversion (E). Upper left: fraction of disadvantageous inequity averse agents in the population. Top center: average wealth per agent. Upper right: distribution of 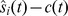 values for steps 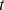 with heterogeneous groups. Lower left: fraction of the total population wealth. Lower right: average age of agents at death.

**Figure 12:**
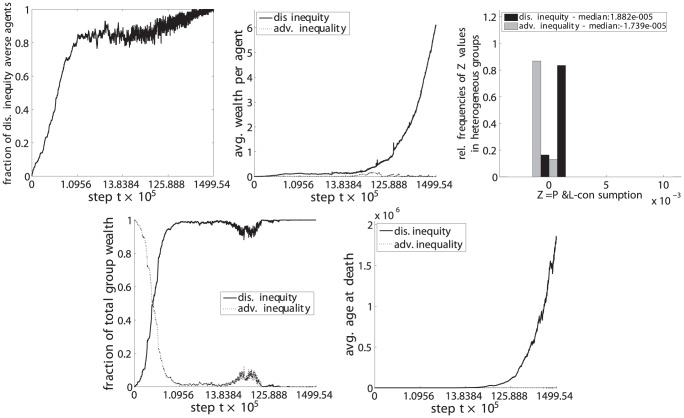
Dis. inequity aversion (C) vs. adv. inequality aversion (F). Upper left: fraction of disadvantageous inequity averse agents in the population. Top center: average wealth per agent. Upper right: distribution of 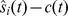 values for steps 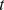 with heterogeneous groups. Lower left: fraction of the total population wealth. Lower right: average age of agents at death.

**Figure 13:**
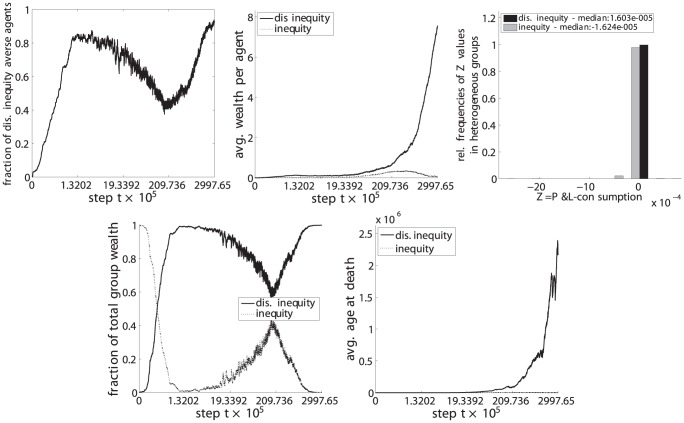
Dis. inequity aversion (C) vs. inequity aversion (A). Upper left: fraction of disadvantageous inequity averse agents in the population. Top center: average wealth per agent. Upper right: distribution of 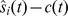 values for steps 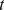 with heterogeneous groups. Lower left: fraction of the total population wealth. Lower right: average age of agents at death.

**Figure 14:**
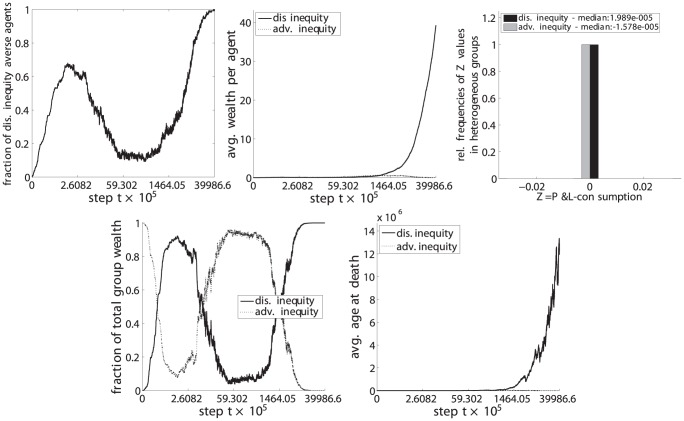
Dis. inequity aversion (C) vs. adv. inequity aversion (D). Upper left: fraction of disadvantageous inequity averse agents in the population. Top center: average wealth per agent. Upper right: distribution of 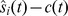 values for steps 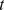 with heterogeneous groups. Lower left: fraction of the total population wealth. Lower right: average age of agents at death.

Running our simulation, we observe that the population of agents becomes always dominated by disadvantageous inequity aversion preferences, independent of which competing variant of self- or other-regarding adaptation dynamics has been seeded at step 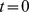. To further understand why we observe this behavior, for each of the six pairwise comparison, we plot in figures 9, 10, 11, 12, 13, 14.

(upper left) the fraction of disadvantageous inequity averse agents compared to the whole population,

(top center) the average wealth per agents in each phenotypic trait class,

(upper right) the relative frequencies of 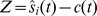, i.e. the P&L minus the consumption, for periods in which groups were heterogeneous, i.e. agents with both phenotypic traits were present in the group,

(lower left) the fraction of the total wealth taken by each phenotypic trait class and.

(lower right) the average age at death for each phenotypic trait class.

The resulting set of a total of 6 pairwise comparisons are depicted in figures 9, 10, 11, 12, 13, and 14, each of them showing the 5 subplots described above. Time steps (x-axis) are indicated in a non linear scale with a total of 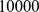 y-value samples taken over the whole simulation steps. The results correspond to 128 system realizations with a total population of 512 agents in 128 groups. The plots show nicely the impact of survival and fertility selection on the population of agents. The indicated metrics in the different subplots conclusively demonstrate how disadvantageous inequity aversion always ends up dominating the population.

Figure 9 (upper left) shows the evolution of the number of disadvantageous inequity averse agents as a fraction of the total number of agents in the population across time. The impact of fertility selection is depicted in figure 9 (top center) with disadvantageous inequity averse agents being able to maintain on average a higher wealth value - also due to the longer lifetimes. Consequently, they are better able to promote their traits in the population. Figure 9 (upper right) shows that, in periods where agents of both phenotypic traits are present, those acting based on disadvantageous inequity aversion clearly outperform self-regarding and selfish-acting agents on the short run. This is indicated by a right-shifted distribution (positive values of P&L-consumption) of the disadvantageous inequity averse agents compared to the left-shifted distribution of those being purely self-regarding and selfish-acting. Disadvantageous inequity averse agents do perform better here because they are less volatile in their adaptations as shown in figure 6 compared to the fluctuating behavior of self regarding agents (see supporting information for details). In this way, agents with adaptation dynamic C suffer from less losses as a result of differences in contributions and punishments, respectively. Additionally, we provide the median value of the two distributions printed in the plot's legend. The fraction of wealth of disadvantageous inequity averse agents compared to the total wealth of the population starts to dominate as can be seen in figure 9 (lower left). This result indicates that disadvantageous inequity averse agents typically invade and take over groups that are heterogeneous with respect to the phenotypic trait 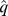. The effect of survival selection is shown in figure 9 (lower right). Groups of disadvantageous inequity averse agents are much more stable and are characterized by, on average, longer lifetimes with correspondingly a lower number of deaths. This makes them being less exposed to cross-over and mutations than compared to purely self-regarding and selfish-acting agents.

Essentially the same results and lines of argumentation hold for the remaining 5 comparisons, ie. inequity aversion (A) vs. disadvantageous inequity aversion (C), inequality aversion (B) vs. (C), advantageous inequity aversion (D) vs. (C), disadvantageous inequality aversion (E) vs. (C) and advantageous inequality aversion (E) vs. (C) as shown in figures 10, 11, 12, 13, and 14.

**Result 3**: *The three effects together (1-higher average wealth, 2-smaller volatility in their adaptation, 3-longer lifetimes) lead to the emergence of disadvantageous inequity aversion and its prepotency compared to the 6 self- and other-regarding preferences listed above.*

These findings together with those reported in the previous section suggest that disadvantageous inequity aversion does not only describe best the punishment behavior observed in lab experiments but moreover is also consistent and coherent with evolutionary dynamics in a competitive, resource limited environment. It seems that evolution inevitably pushes towards the development of a sense for fairness (disadvantageous inequity aversion) in population of adaptive and evolving interacting agents. This likely shapes the contemporary behavior of subjects and provides an explanation for the altruistic behavior observed in modern experiments in the form of the altruistic punishment of defectors.

## Discussion

We developed a simulation model that mimics the long-term co-evolutionary dynamics of different other-regarding preferences such as self-regarding, inequality and inequity aversion. Our approach is one of the first investigations that directly links empirical data from lab experiments to an evolutionary simulation model. In this way, it fills the gap between the existing literature on the theory of evolution applied to cooperation and punishment and the empirical findings from lab experiments and field studies. This is in contrast with most simulation based approaches that deal with the evolution of altruistic behavior and fairness preferences, which focus on the reproduction of stylized facts rather than providing an empirical validation of the computational results as we do. In addition to the existing literature, we allow various fairness preferences, e.g. disadvantageous inequity aversion and purely self-regarding behavior, to co-evolve based on standard evolutionary dynamics. This means that we allow the preferences that determine the underlying behavior to co-evolve rather than having the behavior directly determined by evolutionary dynamics. In contrast to most evolutionary papers, this allows us to abstract from overly simplifying and restricting rules which, of course, are useful and convenient for analytical calculations, but at the same time are less suitable for comparison with empirical observations.

Some of the existing literature, e.g. [82]–[85], lay their focus on the strategic short-term behavior of agents and therefore explicitly anticipate the existence of certain cognitive capabilities in the population. In particular, they presume the ability to memorize the others' behavior from previous interactions. This is different from our approach, which does not explicitly incorporate short-term strategies in the agents' behavior. Another approach explicitly requires the agents to recognize similarities in the behavior of other agents as e.g presented in [86]. This is also different from our approach as, in our model, agents do not have any perception of the willingness to contribute or the propensity to punish of group mates.

A third class of models focus on the voluntary option either to participate or stay away from social dilemma situations [87]–[89]. This allows agents to arrange in groups of similar behaviors. In the simplest case, this is either to cooperate or to defect. However, in our model, agents are forced to participate in the public goods game. Between these two types of models either based on similarity-measures or the option to voluntarily participate in the game, a third class of models is a mixture of the other two, in particular via the inclusion of spatial structures [90]–[93]. Spatial structures allow agents to arrange among like-minded individuals and to stay away, i.e. opt-out, from “unsuitable” or “unfriendly” regions.

Our model does not use any of the assumptions required by the other types of models. It relies essentially on the assumption coming along with the long-term co-evolutionary dynamics of genes and culture. In particular, the indirect evolution of the agents' behavior, which is determined via the co-evolution of their other and self-regarding preferences, is different from the existing approaches. By using this novel approach that closely combines empirical data obtained from laboratory experiments together with a simulation model, we are able to corroborate and complement assumptions made by researchers to tackle the puzzling and ostensibly irrational behavior of humans observed in experiments and field studies.

## Conclusion

We have studied the evolution of fairness preferences in the form of other-regarding behavior and its effect on the origination of altruistic punishment behavior. For this, we have combined empirical results from three public goods experiments together with an evolutionary simulation model. The model borrows ideas from evolutionary biology, behavioral sciences and economics as well as complex system science.

Our first principal result is that, in a evolutionary-competitive resource-limited environment, altruistic punishment behavior can spontaneously emerge in a population of agents who are initially non-punishers, if other-regarding preferences are present. We have shown how this derives from an evolutionary process with adaptation, selection, crossover and mutation for different variants of inequality or inequity aversion.

Our second main result is the identification of disadvantageous inequity aversion as the most relevant underlying mechanism to explain the emergence and the degree of altruistic punishment observed in public goods experiments. This result has been obtained by combining empirical data with an evolutionary simulation model in an innovative way. Our model is able to reproduce quantitatively, without adjustable parameters, the experimental results concerning the level of punishment behavior. This result is of particular importance to substantiate the assumptions made by researchers in order to describe realistic behavior within the framework of rational choice: Humans exhibit other-regarding, and in particular, disadvantageous inequity aversion preferences in their decision process when facing public goods dilemmas with punishment opportunity.

As a third main result, we have demonstrated that disadvantageous inequity aversion is an evolutionary stable preference which dominates pure self-regarding and selfish behavior and also all other analyzed variants of inequity- and inequality aversion in a competitive resource-limited environment. We showed that standard evolutionary dynamics indeed have a built-in affinity to promote other-regarding behavior. This results from the fact that individuals so-to-speak hold each other mutually at bay to first ensure their own survival and second to preferably promote their own genetic and cultural heritage. Other-regarding behavior in the form of disadvantageous inequity aversion is often interpreted as a sense for fairness that serves to explain altruism. However, we find that disadvantageous inequity aversion and altruistic punishment, respectively, are just natural evolutionary consequences in the presence of competitive selection pressure.

In conclusion, we believe that the combination of empirical research and simulation models can provide deeper insights into the evolutionary roots of human behavior. With regard to the often-cited importance of altruistic punishment in promoting cooperation, our simulation model provides a flexible and powerful methodology to answer many remaining research questions including e.g. the influence of group interactions and varying selection pressures. For instance, more realistic set-ups in which agents play several games simultaneously so as to mimic a real life situation can easily be analyzed using our model.

## Supporting Information

Appendix S1 (TEX)

Figure S1
**Dynamics C - dis. inequity aversion.** Evolution of the propensity to punish 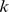 (y-axis) over 5 million time steps (x-axis) (sample taken every 100 steps) resulting from 8 system realizations with a total of 32 agents in 8 groups. The shade of grey indicates (left) the number of deaths per group within 100 simulation steps, (center) if the disadvantageous inequity aversion condition 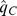 is true or false for a given group and (right) the positiveness/negativeness of the difference between P&L minus the consumption in each period for each of the 32 agents. • The evolutionary barrier visible in figure 6 can also be observed in figure S1 (left), showing the higher rate of deaths/births in the range 0.125<*k*<0.2. This is indicated by a brighter shape of grey. Figure S1 (center) depicts the value of the boolean condition 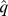 *_C_* on a group level across time, i.e. it quantifies whether all 4 agents per group are satisfied with their realized P&L and the ratio of their contributions in a way that 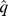 *_C_* becomes *false* (the agents are “happy”). If this applies, no adaptation is performed by the agents, i.e. the agents remain stable in their behavior. For values of *k*<0.125, this is clearly not the case, causing *ki*(*t*) and *mi*(*t*) to continuously evolve. In addition, figure S1 (right) reveals that the agents' survival condition, i.e. P&L-consumption≥0, is only constantly satisfied for levels of the propensity to punish *k*>0.2, while it continuously alternates between positive and negative values below this boundary level.(EPS)

Figure S2
**Dynamics A - inequity aversion.** Evolution of the propensity to punish 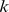 (y-axis) over 5 million time steps (x-axis) (sample taken every 100 steps) resulting from 8 system realizations with a total of 32 agents in 8 groups. The shade of grey indicates (left) the number of deaths per group within 100 simulation steps, (center) if the disadvantageous inequity aversion condition 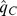 is true or false for a given group and (right) the positiveness/negativeness of the difference between P&L minus the consumption in each period for each of the 32 agents. • Figures S2 to S7 present an overview of the micro-dynamics of the remaining self- and otherregarding preferences (dynamics A,B,D–G). The three subplots show the evolution of the propensity to punish *k* (y-axis) over 5 million time steps (x-axis) (sample shown every 100 steps) resulting from 8 system realizations with a total of 32 agents in 8 groups. In left subplot, the shade of grey indicates the evolution of the wealth. The center subplot depicts if the other-regarding preferences condition 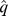 *_A,B,D,E,F,G_* is true or false for a given group. The subplot on the right shows the positiveness/negativeness of the difference between P&L and consumption in each period and for each of the 32 agents. A closer look on the micro-behavior data presented in the figures S2, S3, S4, S5, S6, S7 reveals that the evolutionary dynamics A,B and D–F make agents not to converge to a evolutionary stable and stationary propensity to punish. Figures S2 and S4 reveal that preferences of symmetric inequity aversion and advantageous inequity aversion (dynamics A and D) make agents to quickly explore values *k*>0.125. In contrast to disadvantageous inequity aversion (dynamic C), the conditions 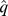 *_A_* and 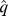 *_D_* can not permanently be resolved to *false* for *k*>0.125. In addition, there exists no unique equilibrium with respect to the fitness, for values of *k* larger than 0.2 as shown in figures S2 (left) and S4 (left). This causes adaptation and evolutionary selection to operate continuously. As a consequence, the populations continue to evolve without achieving a stable evolutionary state. Altruistic punishment also originates in the three analyzed variants of inequality aversion (dynamics B, E and F). Figures S3, S5 and S6 suggest that survival and fertility selection operate in the opposite direction to the inequality aversion preferences, keeping agents away from achieving a potential stable state. While 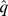 *_B_* can only become *false* for *k*<0.2, agents with *k*≫0.2 outperform those with a smaller propensity to punish as indicated by brighter shades of grey for *k*≫0.2 in figures S3 (left), S5 (left) and S6(left). This leads to an evolutionary dynamic with no statistically stationary behavior and thus results in a heterogeneous population of agents with respect to *k*. Purely self-regarding and selfish-acting agents (dynamic G) do not evolve a significant level of propensity to punish. Figure S7 (left) reveals the existence of a single attraction point *k* = 0 indicated by brighter grey tones towards this value that lasts for the entire simulation. The purely self-regarding and selfish adaptation condition 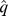 *_G_* does not allow agents to achieve an evolutionary stable state in the range of 0<*k*<0.2, as can be observed in figure S7 (center). As with the inequality aversion preferences, evolutionary selection, with its attraction point at *k* = 0, works in the opposite direction to the adaptation condition G. This results in a population of agents that stay in an evolutionary non-stable range of 0<*k*<0.2.(EPS)

Figure S3
**Dynamics B - inequality aversion with** 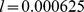 **.** Evolution of the propensity to punish 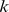 (y-axis) over 5 million time steps (x-axis) (sample taken every 100 steps) resulting from 8 system realizations with a total of 32 agents in 8 groups. The shade of grey indicates (left) the number of deaths per group within 100 simulation steps, (center) if the disadvantageous inequity aversion condition 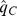 is true or false for a given group and (right) the positiveness/negativeness of the difference between P&L minus the consumption in each period for each of the 32 agents.(EPS)

Figure S4
**Dynamics D - advantageous inequity aversion.** Evolution of the propensity to punish 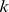 (y-axis) over 5 million time steps (x-axis) (sample taken every 100 steps) resulting from 8 system realizations with a total of 32 agents in 8 groups. The shade of grey indicates (left) the number of deaths per group within 100 simulation steps, (center) if the disadvantageous inequity aversion condition 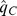 is true or false for a given group and (right) the positiveness/negativeness of the difference between P&L minus the consumption in each period for each of the 32 agents.(EPS)

Figure S5
**Dynamics E - disadvantageous inequality aversion.** Evolution of the propensity to punish 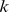 (y-axis) over 5 million time steps (x-axis) (sample taken every 100 steps) resulting from 8 system realizations with a total of 32 agents in 8 groups. The shade of grey indicates (left) the number of deaths per group within 100 simulation steps, (center) if the disadvantageous inequity aversion condition 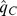 is true or false for a given group and (right) the positiveness/negativeness of the difference between P&L minus the consumption in each period for each of the 32 agents.(EPS)

Figure S6
**Dynamics F - advantageous inequality aversion.** Evolution of the propensity to punish 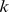 (y-axis) over 5 million time steps (x-axis) (sample taken every 100 steps) resulting from 8 system realizations with a total of 32 agents in 8 groups. The shade of grey indicates (left) the number of deaths per group within 100 simulation steps, (center) if the disadvantageous inequity aversion condition 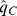 is true or false for a given group and (right) the positiveness/negativeness of the difference between P&L minus the consumption in each period for each of the 32 agents.(EPS)

Figure S7
**Dynamics G - self-regarding agents.** Evolution of the propensity to punish 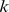 (y-axis) over 5 million time steps (x-axis) (sample taken every 100 steps) resulting from 8 system realizations with a total of 32 agents in 8 groups. The shade of grey indicates (left) the number of deaths per group within 100 simulation steps, (center) if the disadvantageous inequity aversion condition 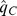 is true or false for a given group and (right) the positiveness/negativeness of the difference between P&L minus the consumption in each period for each of the 32 agents.(EPS)
